# Kinomic Profiling of Electromagnetic Navigational Bronchoscopy Specimens: A New Approach for Personalized Medicine

**DOI:** 10.1371/journal.pone.0116388

**Published:** 2014-12-30

**Authors:** Joshua C. Anderson, Douglas J. Minnich, M. Christian Dobelbower, Alexander J. Denton, Alex M. Dussaq, Ashley N. Gilbert, Timothy D. Rohrbach, Waleed Arafat, Karim Welaya, James A. Bonner, Christopher D. Willey

**Affiliations:** 1 The Department of Radiation Oncology, The University of Alabama at Birmingham, Birmingham, Alabama, United States of America; 2 The Department of Surgery, Division of Cardiothoracic Surgery, The University of Alabama at Birmingham, Birmingham, Alabama, United States of America; 3 The Department of Pathology, The University of Alabama at Birmingham, Birmingham, Alabama, United States of America; 4 The University of Alexandria, Alexandria, Egypt; Pulmonary Medicine, China

## Abstract

**Purpose:**

Researchers are currently seeking relevant lung cancer biomarkers in order to make informed decisions regarding therapeutic selection for patients in so-called “precision medicine.” However, there are challenges to obtaining adequate lung cancer tissue for molecular analyses. Furthermore, current molecular testing of tumors at the genomic or transcriptomic level are very indirect measures of biological response to a drug, particularly for small molecule inhibitors that target kinases. Kinase activity profiling is therefore theorized to be more reflective of *in* *vivo* biology than many current molecular analysis techniques. As a result, this study seeks to prove the feasibility of combining a novel minimally invasive biopsy technique that expands the number of lesions amenable for biopsy with subsequent *ex* *vivo* kinase activity analysis.

**Methods:**

Eight patients with lung lesions of varying location and size were biopsied using the novel electromagnetic navigational bronchoscopy (ENB) technique. Basal kinase activity (kinomic) profiles and *ex* *vivo* interrogation of samples in combination with tyrosine kinase inhibitors erlotinib, crizotinib, and lapatinib were performed by PamStation 12 microarray analysis.

**Results:**

Kinomic profiling qualitatively identified patient specific kinase activity profiles as well as patient and drug specific changes in kinase activity profiles following exposure to inhibitor. Thus, the study has verified the feasibility of ENB as a method for obtaining tissue in adequate quantities for kinomic analysis and has demonstrated the possible use of this tissue acquisition and analysis technique as a method for future study of lung cancer biomarkers.

**Conclusions:**

We demonstrate the feasibility of using ENB-derived biopsies to perform kinase activity assessment in lung cancer patients.

## Introduction

Novel therapies for lung cancer are being tested in both preclinical and clinical settings [1], yet little is known as to how to best select drugs for individual patients. Recent approaches to tackle this problem have used genomic, transcriptomic, and proteomic profiling of tissue [2]–[4] or blood samples [5]. However, these approaches are limited because: 1) They can be time consuming (leading to delay in treatment); 2) Protein and gene expression often show poor concordance (i.e., gene expression does not necessarily predict protein level) [6]; and 3) Genomic and proteomic data are very indirect measures for drug function [7]. This is especially evident with kinase-directed therapies [8], [9] such as tyrosine kinase inhibitors (TKI’s). Unfortunately, almost all clinical studies using TKI’s do not attempt to measure kinase activity, even indirectly [1], [10]. Even innovative trials like the Biomarker-integrated Approaches of Targeted Therapy for Lung Cancer Elimination (BATTLE) Trial [11], do not include kinase activity in their molecular characterization, despite a preponderance of TKI’s being tested. For this reason, current biomarker utilization for predicting response to molecularly targeted agents has been limited to a very small list of mutated kinases. EGFR activating mutations and the EML4-ALK genetic translocation, the two most prominent examples that predict response to specific TKI’s, are, unfortunately, fairly rare [12]. Thus, identifying clear targetable pathways has been possible for only a small minority of patients. Complicating matters is the fact that obtaining adequate tissue for molecular testing can be difficult [2], [13]–[15]. For these reasons, a biomarker discovery effort that couples an innovative surgical approach with kinase activity (“kinomics”) evaluation was sought for lung cancer patients.

**Figure 1:**
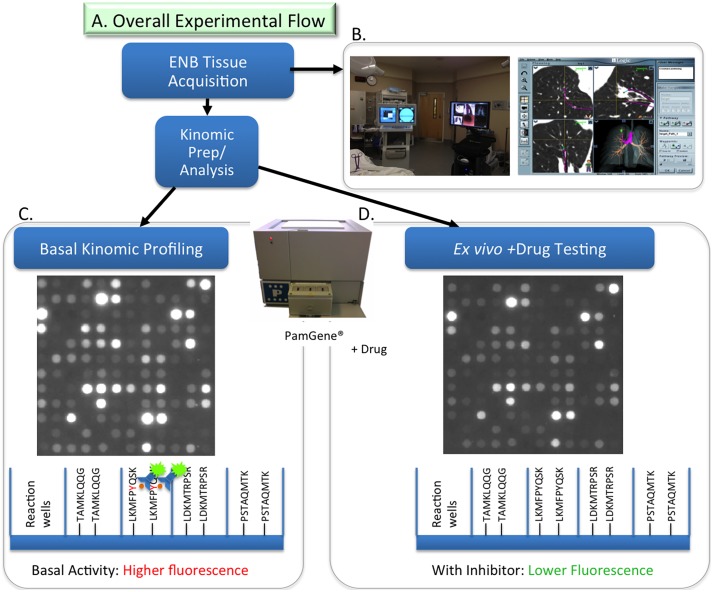
Kinomic platform and Electromagnetic Navigation Bronchoscopy. (A) Overall experimental flow with a (B) Representative in-procedure display of ENB and a schematic of PamChip assay used to measure basal kinomic activity displayed as (C) raw array picture of the 144 phosphorylatable peptides and (D) phosphorylation changes with drug treatment displayed with illustration of comparative fluorescent detection below.

Kinomic profiling refers to the determination of global kinase activity in a specimen and is distinct from genomic and proteomic methods because it determines changes in biological activity, not just the presence of a gene, transcript, or protein [16]. Our lab utilizes the PamStation 12 (PamGene, B.V., Hertogenbosch, Netherlands), a fluorescent assay platform (Fig. 1) requiring very small quantities of lysate that can measure the ability of active kinases in a specimen to phosphorylate specific peptides, imprinted on multiplex arrays, in real time with kinetic evaluation allowing for kinomic profiling of cells and tumor tissue [17], [18].

As mentioned earlier, one of the limitations commonly faced in the clinic relates to the difficulty in obtaining adequate tissue for molecular analysis [2], [13], [19]. Fine needle aspirates of lung lesions may provide histologic diagnosis and have shown some utility in specific molecular testing (ALK fusion and EGFR mutation detection) [20], [21], but are often inadequate in quantity for more extensive molecular profiling, including kinomics. Heretofore, to obtain larger amounts of material required: a) that the lesion be resectable (i.e., early stage in a medically fit patient); b) that the tumor be large; c) that the tumor be located in proximal portions of the bronchial tree; and/or d) that the patient undergo more invasive procedures. This is particularly evident in clinical trials for lung cancer where obtaining fresh-frozen tissue (a requirement for kinomics and several other microarray platforms) has been quite limiting.

Electromagnetic navigational bronchoscopy (ENB) can potentially overcome some of these limitations by more reliably obtaining diagnostic material from almost any location in the lungs in sufficient quantities to allow for molecular testing [22]. ENB (Fig. 1) utilizes three key components to generate a “virtual” bronchoscopy: 1) a specially formatted computed tomography (CT) scan; 2) an electromagnetic plate and chest wall sensors to monitor patient movement with respiration; and 3) an extended working channel with a locatable guide. The locatable guide allows the physician to navigate to the target lesion and biopsy specimens are taken under fluoroscopic visualization using forceps in a manner that has been shown to increase clinical yield [22]. In the present study, we tested the feasibility of using ENB-derived tissue specimens with kinomic profiling for detecting biological activity in lung lesions.

## Methods

### Ethics

This study was conducted in accordance with the Declaration of Helsinki and was approved by University of Alabama at Birmingham (UAB) Institutional Review Board (IRB# X110411014). Written informed consent was obtained from all patients.

### Electromagnetic Navigational Bronchoscopy and Biopsy Procedure

ENB utilizes a specially formatted CT scan to create a virtual bronchoscopy. During the procedure the patient lies on an electromagnetic plate and chest wall sensors monitor patient movement with respiration. A standard video bronchoscope is used with a 3 mm working channel. An extended working channel and a locatable guide are passed through the bronchoscope’s working channel. The tip of the locatable guide is sensed in the electromagnetic field. Registration points are identified to match the virtual images to the patient's anatomy. The locatable guide is then navigated to the target lesion and biopsy specimens are taken under fluoroscopic visualization. For this study, the tissue specimens were collected in cryovials and immediately flash frozen in liquid nitrogen in the operating suite. These specimens were transferred directly to the UAB Kinome Core (www.kinomecore.com) and stored at −80 C until the kinomic assay was performed. During the same ENB procedure, tumor specimen material was collected and provided to UAB Pathology when clinically indicated. In some cases, the patient already had a tissue diagnosis and underwent fiducial implantation for facilitation of stereotactic body radiation therapy treatment planning and delivery. These fiducials were placed after obtaining any biopsy material.

### Sample Collection and Processing for Kinomic Profiling

Frozen tissue for each tumor was processed at 4°C for kinomic profiling using eppendorf tube pestle grinding followed by lysis in M-PER lysis buffer with protease and phosphatase inhibitors per our standard protocol [23]. For those patients without a prior cancer diagnosis, a simultaneously collected tissue specimen was also sent to pathology for evaluation. Protein quantitation was performed using standard BCA reaction assay. Based on total protein available for each specimen, 5–10 µg of protein were combined with kinase buffer, ATP, and fluorescently labeled anti-PY20 antibodies per UAB Kinome Core standard operating procedure and then loaded into each well of the tyrosine (PTK) PamChips. The samples were run on the PamStation 12 kinomics workstation (PamGene International, ‘s-Hertogenbosch, The Netherlands) using the standard PTK PamChip protocol using Evolve12 Software (v. 1.5) as previously published [23]. As lysates were pumped through the array, images were captured and followed by analysis and quantitation performed using BioNavigator v. 5.1 (PamGene). Samples were excluded from subsequent *ex* *vivo* drug testing if they had a) insufficient protein quantity, b) excessive temperature variation, c) insufficient basal kinase activity. Western blotting to measure relative levels of housekeeping proteins and total phospho-tyrosine was completed using 10% SDS-PAGE gels, and transferred to Immobilon PVDF membranes (Merck, Darmstadt, Germany) with 20 ug of sample loaded per lane, and detection with 1∶4000 anti-phospho-tyrosine-HRP (Santa Cruz Biotechnology, Santa Cruz, CA, scbt-508), 1∶5000 mouse anti-GAPDH and rabbit anti-actin (Santa Cruz, scbt-51907 and scbt-1616) with horseradish-peroxidase conjugated goat anti-mouse, and donkey anti-rabbit antibodies (Jackson ImmunoResearch, West Grove, PA, 115-035-166 and 711-035-153).

### *Ex Vivo* Drug Testing

*Ex vivo* kinomics testing was performed using the small molecule kinase inhibitors, lapatinib, crizotinib, and erlotinib purchased from LC Laboratories (Woburn, MA). *Ex vivo* testing [24] involved pre-mixing 25 µL aliquots of protein extract (5 µg) from each tumor lysate prepared as above in separate tubes with each drug at 20 nM, 0.5 µM, or 20 µM or vehicle (dimethylsulfoxide, DMSO), incubating for 15 mins at 4°C, adding kinase assay mix (Master Mix, PamGene) and applying the mixture to an array of the PTK chip. Three chips (12 wells) were loaded with 35 µL of the mixture and inserted into the PamStation where kinase activity and image capture took place over one hour. The PamChip protocol and analysis using Evolve12 and BioNavigator were done as before with the basal analysis.

### Statistical Analysis

Primary analysis was performed using BioNavigator software v. 5.1 (PamGene).

Kinase activity data were produced to correspond to the phosphorylatable-peptide specific reaction per ‘spot’ on the PamChip. Raw signal intensity data per each of the 144 spots was captured over multiple 50 ms exposures sequentially as lysates were pumped through the array, and then over multiple exposure times (10,20,50,100,200 ms) after lysates were rinsed off. These values were converted to slopes of intensity by exposure time, and slopes were multiplied by 100 and log2 transformed for visualization and are labeled “log signal”. Heatmaps of log signal data were plotted in color corresponding with signal intensity (basal conditions) or with log change from control (drug response). Unsupervised hierarchical clustering of kinomic profiles for the samples was done using a Euclidean distance, complete linkage R-script within BioNavigator. Variance filtering was also performed on the log signal transformed data with deviation from peptide experimental mean per peptide in basal conditions. Dose-response heatmaps, are displayed as the log ratio change from a DMSO control. Selected peptides were plotted as raw kinetic values (“prewash signal”) over multiple 50 ms exposures to demonstrate specific treatment-altered phosphorylation curves. A publically available kinomic analysis tool containing the experimental data can be found at http://kinome.github.io/public.

## Results

### Patient Characteristics

**Figure 2:**
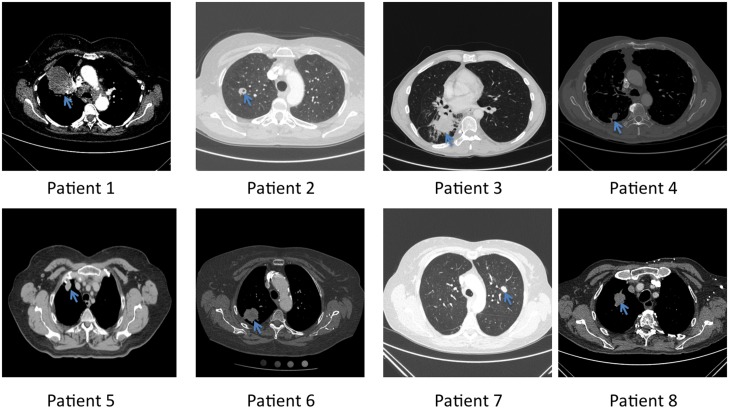
Patients’ computed tomography (CT) scans. CT scans show representative slices of the lung lesions (blue arrow) prior to ENB. Patient numbers correspond to those listed in Table 1.

**Figure 3:**
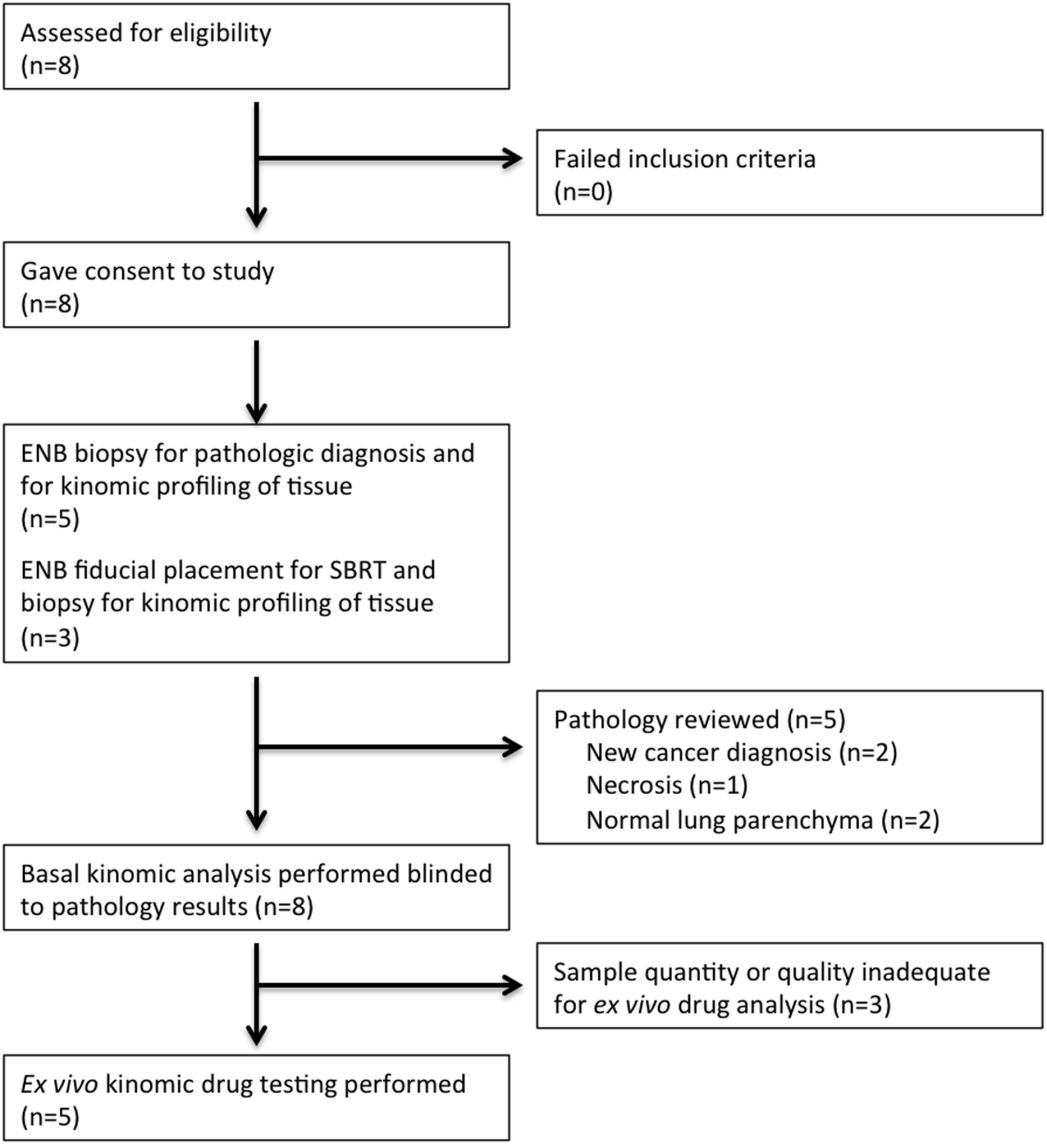
CONSORT diagram on patient enrollment.

In this feasibility study, we consented and enrolled eight patients for kinomic testing of ENB-derived material (Table 1), and all patients were seen and evaluated for this study between October 14, 2011 and May 7, 2013. Eligibility required that each patient show radiographic evidence for a lung mass lesion (Shown in Fig. 2), and be scheduled to undergo an ENB procedure. The majority of patients were women and the median age was 70.5 years of age (range, 49–82) (Table 1). ENB-derived specimens were collected and flash frozen for kinomic analysis within the operating room and were transferred at −80°C to the UAB Kinome Core within 5 minutes. Fig. 3 details the Consolidated Standard of Reporting Trials (CONSORT) schematic showing the flow of the 8 patients who were consented for the study. Of the 8 patients, 5 patients had ENB-biopsy material sent to pathology while the remaining 3 patients had prior tissue diagnosis of non-small cell lung cancer (NSCLC) and underwent the ENB procedure solely for fiducial marker placement related to planned stereotactic body radiation therapy (SBRT). As such, 3 patients did not have simultaneous pathologic evaluation of the ENB-derived specimen. Pathological evaluation yielded malignancy, normal lung, and necrosis in the numbers shown in Table 1. Of note, one patient (#8) whose whole biopsy yielded normal lung parenchyma was later shown on core biopsy to have squamous cell carcinoma. Kinomic profiling was performed in a pathology-blinded fashion.

**Table 1:** Patient characteristics and tumor evaluation.

Patient	Sex	Age	Pathology of Biopsy	Comments
1	F	77	NSCLC, MD-PD, favor adenocarcinoma	
2	F	66	Necrosis, no viable neoplasm identified	
3	M	49	Not obtained	Prior NSCLC diagnosis, fiducial placed for SBRT
4	M	73	Small cell carcinoma	
5	F	73	Not obtained	Prior NSCLC diagnosis, fiducial placed for SBRT
6	F	68	Not obtained	Prior adenocarcinoma diagnosis, fiducial placed for SBRT
7	F	52	Alveolar tissue and cartilage fragments	Prior history of C/L lung adenocarcinoma
8	M	82	Lung parenchyma with no evidence of malignancy	Core biopsy later showed squamous cell carcinoma
**Median:**		70.5		
**Range:**		49–82		

M-male; F-female; NSCLC-non small cell lung cancer; MD-PD-moderately differentiated to poorly differentiated; SBRT-stereotactic body radiotherapy.

### Tissue Quality

Protein quantification of the samples indicated that the tissue specimens were of sufficient size to perform kinomic testing. Typical protein yield for an ENB biopsy sample obtained by micro-forceps was ∼378 µg/mL in approximately 150 µL of total volume. Since kinomic testing requires between 1–5 µg of total protein depending on array type (the serine-threonine kinome [STK] chip requiring 1 µg and the tyrosine kinome [PTK] chip requiring 5 µg), the samples obtained were more than adequate. Variation in loading control protein expression did not always linearly correlate with measured total protein concentrations. Although there was adequate protein, patients 2, 3, and 5 were not suitable for *ex* *vivo* drug testing due to at least one of the exclusion criteria listed above. Pathologic evaluation of patient 2’s specimen indicated that the area biopsied was necrotic which would explain the relatively low level of kinase activity. Unfortunately, the specimens obtained from patients 3 and 5 were not evaluated by pathology due to prior diagnosis of non-small cell lung carcinoma (NSCLC). Of note, patient 3 had previously received definitive chemoradiation to the region but had evidence of progression by CT. Nevertheless, we cannot comment on the viability status of those specimens.

### Basal Kinomic Profiles

**Figure 4:**
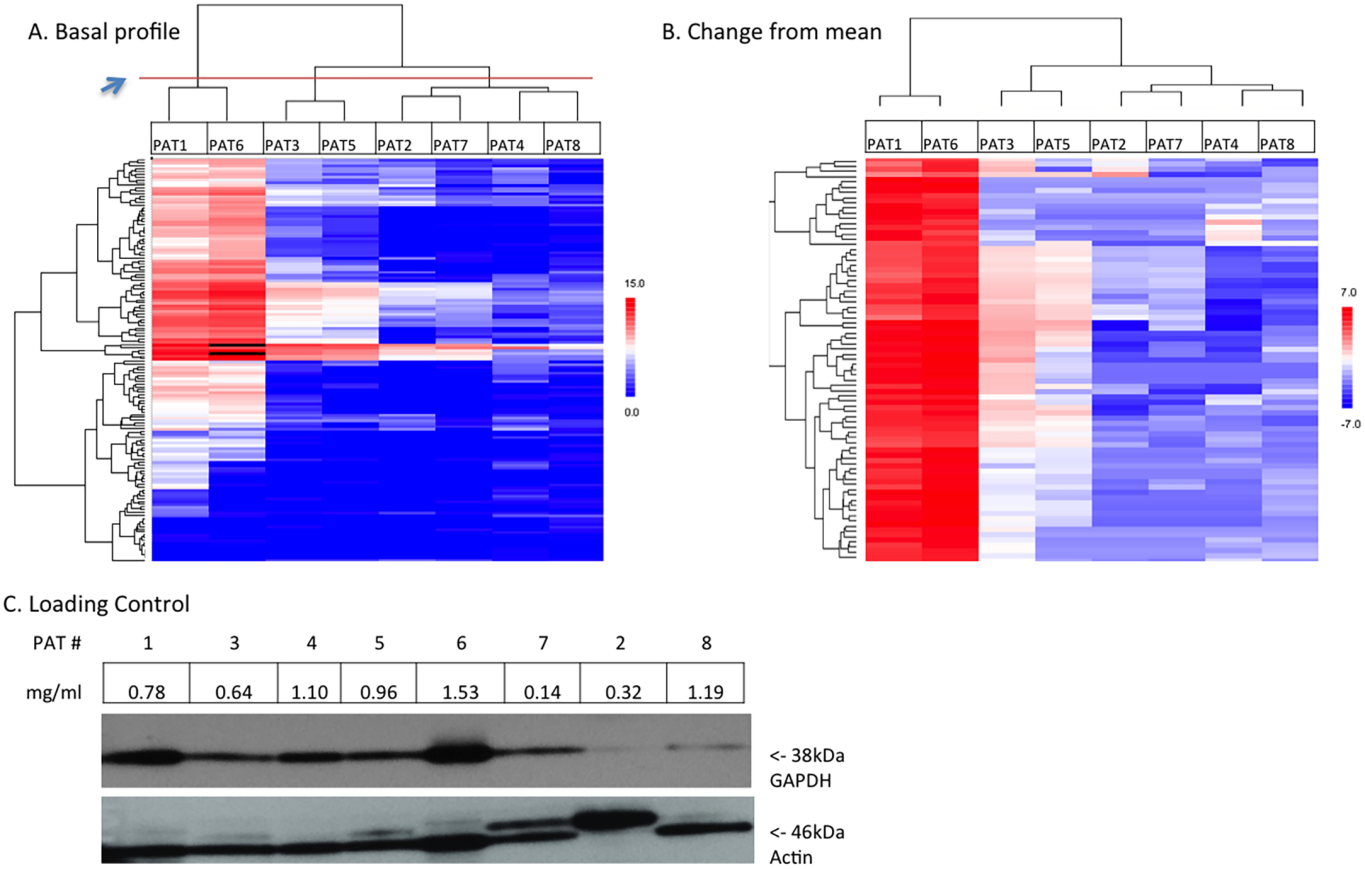
Basal kinomic activity profiles. Unsupervised hierarchical clustering of basal (untreated) tyrosine kinomic profiles displaying log transformed slope-exposure for (**A**) all 144 peptides and (**B**) as change from sample mean and filtered for variance >1. Red in (A) indicates relative increased signal and in (B) indicates an increase from sample mean. Blue indicates the opposite. Blue arrowhead points to red line denoting dendrogram separation. (**C**) Western blotting of GAPDH and Actin is shown with sample concentration indicated for each patient.

The basal kinomic profiles of all 8 patients are shown as unsupervised hierarchically clustered heatmaps of log signal intensity for each phosphosubstrate (Fig. 4; individual peptide phosphorylation curves can be visualized via http://kinome.github.io/public). All 8 patients were analyzed as this was a feasibility study, though only 2 of the 5 patients that had simultaneous pathology review had viable cancer cells. There was a large variation in mean log signal intensity among the samples consistent with the variety of tissues obtained (e.g., normal lung parenchyma, necrosis, and carcinoma). We measured a maximum peptide log signal variance of 25.86, with mean peptide variance of 9.777. The standard deviation from the experimental all-peptide mean (a surrogate for alterations of global kinase activity on the chip) was 2.54 with the highest signal sample having a mean log ratio change from the experimental mean of +4.13(PAT1) and the lowest had a −2.02 (PAT7) log ratio change. Technical variance (aliquoted from same master-sample, run on different arrays and chips) was measured across all peptides (0 values excluded) at 2.7% CV. This was inline with previous experiments where we had seen median technical and biological variance in controlled cell lysates measured at 4.2% and 4.6% CV (data not shown).

Unsupervised hierarchical clustering identified 3 major clusters when the dendrogram was cut at the level of the red line (blue arrow in Fig. 4A). Samples are clustered by patient along the x-axis, and peptides along the y-axis. Two of these clusters contained 4 NSCLC tumors (including one adenocarcinoma; PATs -1, -3, -5, -6) and the other cluster contained no NSCLC (1 necrotic sample, 2 normal tissue samples, and 1 small cell carcinoma [SCC] sample). Fig. 4C displays BCA calculated protein concentration per sample and actin and GAPDH expression levels. Variance filtering for peptides with high variance (>10) was used to further segregate and cluster in the heatmap in Fig. 4B, where peptides are colored by deviation from experimental mean, clustering in similar groups as before, and identifying very high relative log signal in variant peptides in PAT1 and PAT6 as compared to the others. Thus, in general, confirmed malignancies on pathological evaluation had higher signal intensity with samples from patient 1 and 6 being the highest (confirmed by anti-phosphotyrosine Western blot for patients 1 and 6 is shown in S1 Fig.).

### *Ex vivo* Kinase Inhibitor Testing

**Figure 5:**
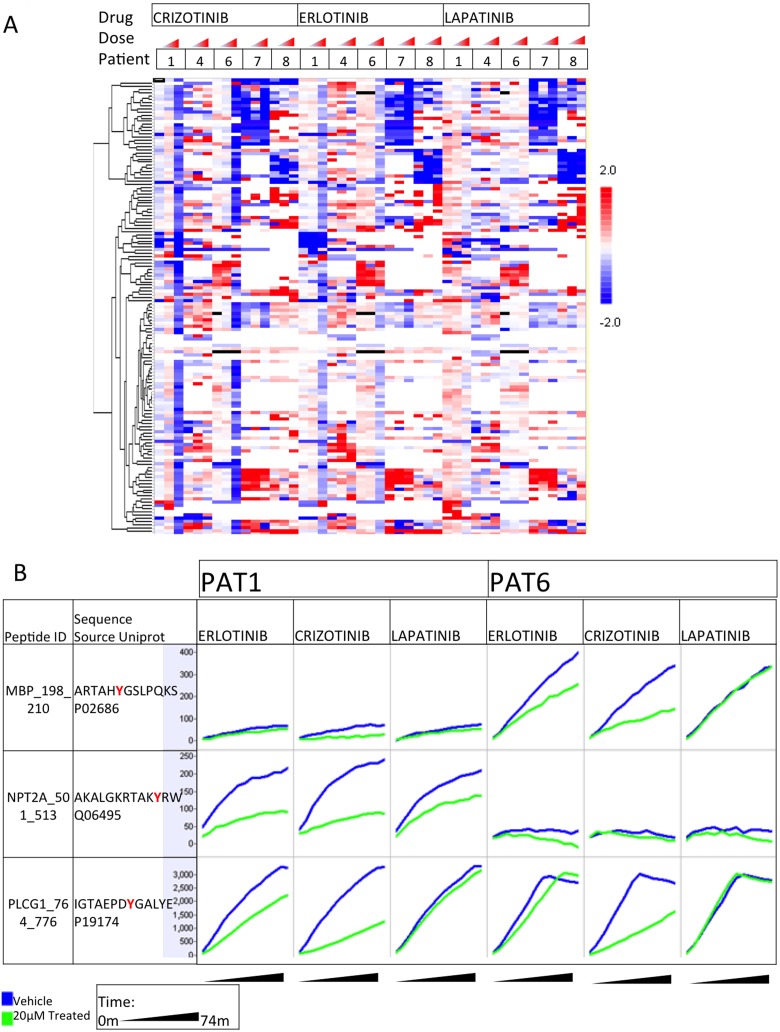
*Ex vivo* drug response profile. Displays *ex* *vivo* drug response profiles as (A) a heatmap of kinase activity (log signal values) change from untreated, clustered by row, of altered phosphopeptides per patient, per dose at 20 nM, 0.5 µM or 20 µM. (B) *Ex vivo* prewash kinetic peptide phosphorylation (y axis per cell) over time (x axis per cell) of selected peptides in the selected samples, in response to indicated drugs at 20 µM. Blue lines denote untreated, and green lines indicate treated phosphorylation curves.

One of the major goals of this project was to determine whether or not ENB-derived lung tumor specimens could be “biologically-interrogated” with small molecule inhibitors. Because the kinomic platform used in this study directly measures kinase activity, we could spike in small molecule kinase inhibitors to determine the change in observed kinomic profiles for each specimen, potentially providing drug response information prior to therapy. This “*ex* *vivo*” profiling of lung tumor specimens collected by bronchoscopic means has not been reported to our knowledge. Therefore, the samples demonstrating adequate signal intensity were treated with either a vehicle control, or increasing doses of the small molecule kinase inhibitors, crizotinib, erlotinib, or lapatinib at time of kinomic evaluation. Hierarchical clustered heatmaps for log-transformed signal intensities of the phosphosubstrates are shown in Fig. 5A. Interestingly, the drug response data showed a more global attenuation of kinase signaling by crizotinib treatment, but only for patients 1 and 6, tumors that also had the highest basal kinase activity (mean log signal of all peptides). On the other hand, lapatinib treatment had a very modest impact on kinase signaling. To further demonstrate the discrimination possible with *ex* *vivo* profiling, the entire kinetic profiles of the kinase assay are shown for 3 of the phosphosubstrate probes for patients 1 and 6 (Fig. 5B). Whereas the MBP_198_210 probe showed minimal increase in activity over the course of the assay for both DMSO and drug treatment in patient 1’s specimen, in patient 6’s tumor, this probe showed a time dependent increase in phosphorylation intensity for vehicle control (DMSO) treated specimens that was attenuated by both erlotinib and crizotinib, but not by lapatinib. Alternatively, the NPT2A_501_513 probe was phosphorylated in patient 1’s tumor and could be attenuated by all three drugs while patient 6’s tumor showed no change in phosphorylation under any condition. Finally, the PLCG1_764_776 probe showed similar increase in phosphorylation for both patients, but only crizotinib could attenuate this in both patients. Therefore, we feel that kinomic testing in this manner can provide unique, therapeutic-specific information for lung cancer patients.

## Discussion

Adequate tumor tissue quantities for molecular analysis are a known problem in the search for patient specific predictors of prognosis and treatment response prediction [2], and the problem is most salient in patients whose lesions are not accessible by conventional biopsy techniques. As can be seen in Fig. 2, the peripheral location of some of these lesions would render them inaccessible to conventional bronchoscopy; however, the ENB technique described above allowed for the retrieval of adequate amounts of tissue for pathologic diagnosis and kinomic analysis, and the fresh freezing technique did not eliminate the kinase profile. It is possible that kinase signaling is changed to some extent through the ENB collection, or through the liquid nitrogen flash freezing process, (essentially stopping cellular kinase activity, proteolysis as well and other cellular processes). However, it was impractical to collect and analyze fresh tissues without freezing them to directly test this, as variation in time from collection to analysis could not be controlled, and further, ourselves and others have successfully interrogated kinase activity in flash frozen tissue from cellular, human, and animal tissues [25]–[27].

Further refinement of this technique will involve optimizing patient selection and improving the yield of acquiring tumor samples, as well as comparing a larger cohort of samples against tissues acquired using a standard biopsy technique or even better, from patients who ultimately undergo subsequent surgical resection. In this way, we would be able to explore congruency between ENB-biopsy material and primary resection specimen and will allow us to better determine the impact of tumor heterogeneity on kinomic activity. One approach that is currently being investigated is the use of confocal laser endomicroscopy (Cellvizio system, Mauna Kea Technologies, Paris, France) during bronchoscopy procedures, including ENB. This “optical biopsy” can improve diagnostic yields since neoplastic surface mucosa can be more readily distinguished from non-neoplastic mucosa [28]. This may be especially useful in patients who will not be undergoing surgical resection of lesions.

The basal heatmap (Fig. 4) intimates the possible utility of the combination of ENB followed by kinomic analysis. First, tissue was obtained in adequate quantities to produce this type of profiling data. Additionally, the patients appear to show distinct differences in their basal profiles; of note, the unsupervised analysis can be interpreted to have clustered NSCLC as opposed to all other tissue types despite the small sample numbers (Fig. 4A). This clustering suggests that kinomic analysis of ENB derived tissue will allow for patient and tumor specific analyses as further knowledge of the lung cancer kinome and its relevance to prognosis and treatment response is acquired. As an example, the type of kinomic activity data shown in Figs. 4 and 5 have already been shown in rectal cancer to be amenable to the creation of an accurate model to predict drug and radiation treatment response [24], and a similar approach to lung cancer is likely feasible.

Furthermore, *ex* *vivo* small molecule inhibitor testing (Fig. 5A) appears to show inhibition patterns that are both patient and drug specific. For example, note the general inhibition seen as the mostly blue column at the highest dose of erlotinib and crizotinib in patients 1 and 6 (Fig. 5A). Additionally, erlotinib appears to show clustering of more specific peptide inhibition in patients 1, 7, and 8 but not patients 4 and 6 (Fig. 5A). Patient 7 appears to show a specific inhibition pattern seen at all concentrations and for all three of the inhibitors tested. The distinct patient and drug-specific inhibition of kinase activity on single peptides is further visualized in Fig. 5B. For example, note how kinase activity on PLCG1_764_776 appears to be inhibited similarly by crizotinib in both patients 1 and 6; conversely, erlotinib inhibition of the phosphorylation of this peptide seems stronger in patient 1. S2 Fig. shows MetaCore generated network maps generated for these data sets. While the importance of these particular findings remains unknown for the patients included in this study, the findings demonstrate that this sort of patient specific and drug specific kinomic response data is possible in the small amount of tissue derived from ENB. Thus, the combination of ENB and kinomic analysis will likely be useful in the discovery of relevant biomarkers for lung cancer and in the creation of models and tests to predict tumor response to various small molecule inhibitors.

Limitations of this study are primarily related to the small patient population and incomplete pathological evaluation. For example, there are inadequate numbers of known diseased tissue (and subtypes of diseased tissue) versus normal tissue to allow for confident identification of relevant tumor markers. Additionally, there are inadequate numbers of patients to build meaningful models predictive of treatment response. Since some patients did not have a specimen sent for pathological evaluation, we also cannot determine whether the samples with low global kinomic activity was due to the presence of non-viable tissue. As with standard biopsy collection methods, a limitation in molecular analysis is that tumors are highly heterogeneous, even within a single solid tumor, therefore making extrapolations from single biopsies to the entire tumor’s biology is difficult. Comparative multi-site intra-tumor sampling analyses and comparison between ENB-derived tissues and standard biopsy derived tissues in the same patient are generally not feasible from a clinical standpoint. Although this study provides conceptual proof of the feasibility of prospective kinomic analysis of ENB derived tissue, further research will be necessary to investigate the application of this technique to the “precision medicine” landscape of lung cancer.

## Conclusion

This study demonstrates the feasibility of kinase activity microarray testing of tissue derived by electromagnetic navigational bronchoscopy.

## Supporting Information

S1 Fig Anti-phosphotyrosine Western blot was performed for total protein lysates from patient #1 and #6 (PAT1 and PAT6) using 1∶3000 HRP-conjugated anti-phosphotyrosine antibody with anti-actin as a loading control.
(TIF)

S2 Fig Top upstream kinases and network maps for each of the 3 differentially phosphorylated substrates shown in Fig. 5. Peptides are listed (**A–C**) above tables of their respective Kinexus identified and ranked upstream ‘Kinases’ and Uniprot ID’s. The percentage of times a kinase was present in a top-ten list upstream of a phosphorylatable residue in the substrate is listed as ’%hits’. These upstream kinases (red circles on network maps) identified were uploaded by Uniprot ID to GeneGo MetaCore for network modeling (**D–F**, Djikstras shortest paths, with two steps max between input kinases).
(TIF)
