# Correction: Kinomic Profiling of Electromagnetic Navigational Bronchoscopy Specimens: A New Approach for Personalized Medicine

**DOI:** 10.1371/journal.pone.0161986

**Published:** 2016-08-24

**Authors:** Joshua C. Anderson, Douglas J. Minnich, M. Christian Dobelbower, Alexander J. Denton, Alex M. Dussaq, Ashley N. Gilbert, Timothy D. Rohrbach, Waleed Arafat, Karim Welaya, James A. Bonner, Christopher D. Willey

The affiliation for the eighth author is incorrect. Waleed Arafat is not affiliated with #3 but with #4 The University of Alexandria, Alexandria, Egypt.
