# Supplementary figures and images for: Kinomic Profiling of Electromagnetic Navigational Bronchoscopy Specimens: A New Approach for Personalized Medicine
Source: PLoS One. 2014 Dec 30;9(12):e116388. doi: 10.1371/journal.pone.0116388 (PMC4280210; doi:10.1371/journal.pone.0116388)

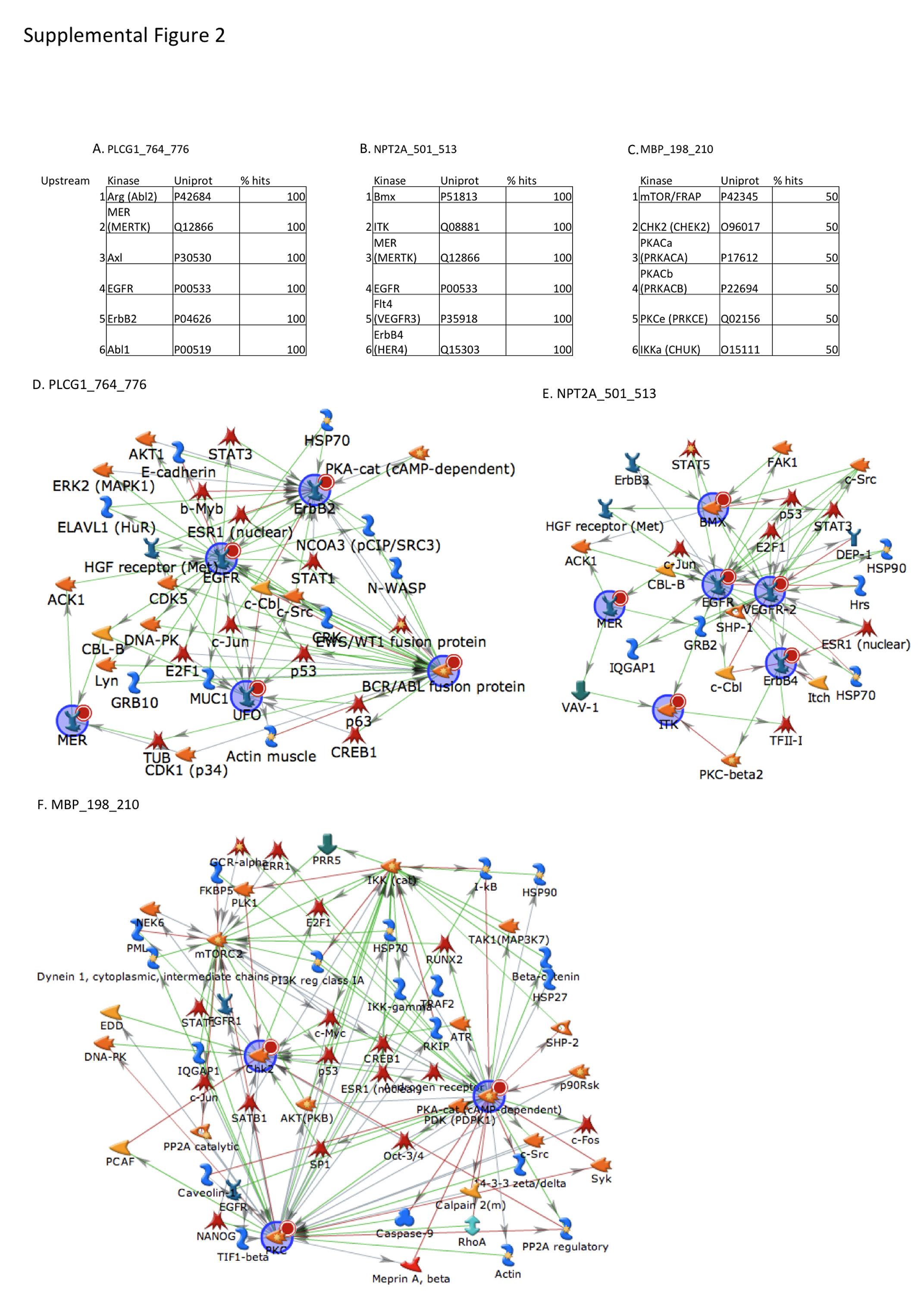

Supplement: S2 Fig — Top upstream kinases and network maps for each of the 3 differentially phosphorylated substrates shown in Fig. 5. Peptides are listed (A–C) above tables of their respective Kinexus identified and ranked upstream ‘Kinases’ and Uniprot ID’s. The percentage of times a kinase was present in a top-ten list upstream of a phosphorylatable residue in the substrate is listed as ’%hits’. These upstream kinases (red circles on network maps) identified were uploaded by Uniprot ID to GeneGo MetaCore for network modeling (D–F, Djikstras shortest paths, with two steps max between input kinases). (TIF) [file pone.0116388.s002.tif]
